# Prognostic implication of p27^Kip1^, Skp2 and Cks1 expression in renal cell carcinoma: a tissue microarray study

**DOI:** 10.1186/1756-9966-27-51

**Published:** 2008-10-15

**Authors:** Zheng Liu, Qiang Fu, Jiaju Lv, Facheng Wang, Kejia Ding

**Affiliations:** 1 Department of Urology, Shandong Provincial Hospital, Shandong University, 324# Jingwu Weiqi road, Jinan, 250021, PR China

## Abstract

**Background:**

p27^Kip1 ^plays a major role as a negative regulator of the cell cycle. The regulation of p27^Kip1 ^degradation is mediated by its specific ubiquitin ligase subunits S-phase kinase protein (Skp) 2 and cyclin-dependent kinase subunit (Cks) 1. However, little is known regarding the prognostic utility of p27^Kip1^, Skp2 and Cks1 expression in renal cell carcinoma.

**Methods:**

Immunohistochemistry was performed for p27^Kip1^, Skp2 and Cks1 in tissue microarrays of 482 renal cell carcinomas with follow-up. The data were correlated with clinicopathological features. The univariate and multivariate survival analyses were also performed to determine their prognostic significance.

**Results:**

Immunoreactivity of p27^Kip1^, Skp2 and Cks1 was noted in 357, 71 and 82 patients, respectively. Skp2 and Cks1 expression were not noted in chromophobe cancers. A strong correlation was found between Skp2 and Cks1 expression (P < 0.001), both of which were inversely related to p27^Kip1 ^levels (P = 0.006 and P < 0.001), especially in primary and clear-cell cancers. Low p27^Kip1 ^expression and Skp2 expression were correlated with larger tumor size and higher stage, as well as tumor necrosis. Cks1 expression was only correlated with tumor size. In univariate analysis, low p27^Kip1 ^expression, Skp2 and Cks1 expression were all associated with a poor prognosis, while in multivariate analysis, only low p27^Kip1 ^expression were independent prognostic factors for both cancer specific survival and recurrence-free survival in patients with RCC.

**Conclusion:**

Our results suggest that immunohistochemical expression levels of p27^Kip1^, Skp2 and Cks1 may serve as markers with prognostic value in renal cell carcinoma.

## Background

Renal cell carcinoma (RCC) is the most common malignancy in adult kidney, with 30,000 new cases per year in the U.S. and 20,000 cases in the European Union [1]. Over the last 20 years, the incidence of renal cell carcinoma in the two regions has increased by 30% [2]. Though the number of RCC cases in Asian is still unknown, publications in this regard have suggested a tendency of annual increase. In light of this situation, predicting the prognosis of RCC patients becomes essential for planning and optimizing treatment strategies. The prognosis of RCC is usually affected by such factors as performance status, pathological stage, tumor size, nuclear grading, and microscopic tumor necrosis. Yet, the accuracy of the traditional clinical and histologic markers is still unsatisfactory in certain clinical settings. There lies the possibility that biologic markers, which have associated with tumor progression, could serve as accurate prognostic markers or targets for specific intervention.

As the alteration of cell cycle is a hallmark of cancer, proteins that are intimately involved in cell cycle regulation are of particular interest. The cell cycle progression is largely dependent on cyclins and cyclin-dependent kinases (Cdks) [3]. Cdks are regulated by Cdk inhibitors, including the INK4 family and the Cip/Kip family. The p27, a member of the latter (p27/Kip1), negatively regulates cell cycle by inactivating cyclin-CDK complex and preventing the transition from G1 to S phase. The degradation of p27 stimulates the activity of Cdk2/cyclin E and Cdk2/cyclin A to promote cell proliferation. Recent evidence also suggests that p27^Kip1 ^is a putative tumor suppressor, thus the loss of p27^Kip1 ^may lead to the uncontrolled proliferation of malignant cells [4]. Recently, reduced expression of p27^Kip1 ^protein has been proved to be highly associated with tumor progression and poor prognosis in various malignant diseases [5]. However, downregulation of p27^Kip1 ^mRNA is rarely observed in human cancers [6]. Instead, the decrease in p27^Kip1 ^levels results mainly from ubiquitin-mediated proteolysis, regulated by the F-box protein SKP2(S-phase kinase-associated protein 2), and its cofactor, Cks1 [7]. SKP2 is an important component of the Skp1-Cullin-F-box protein (SCF) complex, which functions as the main rate-limiting regulator for the degradation of p27^Kip1^. Hence, overexpression of Skp2 may lead to cell-cycle progression. Recent studies have also found that Skp2 may modulate invasion of cancer cells independent of p27 degradation [8]. Cks1 is a member of the highly conserved Cks/Suc1 proteins family, which confers an allosteric change in Skp2 to increase its affinity to phosphorylated p27^Kip1 ^substrate [9,10]. Therefore, p27^Kip1 ^degradation is dependent upon the accumulation of Skp2 and Cks1 as well as the rise in cyclin E. Recently, the expression levels of p27^Kip1^, Skp2 and Cks1 were shown to be highly associated with prognosis in a variety of cancers [10-13].

To date, very few studies have addressed the prognostic role of P27^Kip1^and Skp2 in renal cell carcinoma. And no study has elucidated the roles of Cks1 in RCCs. By using tissue microarray, we therefore aimed at analyzing the immunohistochemical expression patterns of p27^Kip1^, Skp2, and Cks1 proteins, and their associations with clinical and pathologic factors, as well as the prognostic implications.

## Methods

### Patients and specimens

Our study cohort consisted of 482 patients who underwent radical or partial nephrectomy for RCC at the Shandong Provincial Hospital between 1993 and 2005. As approved by the ethical committees of Shandong Provincial Hospital, Formalin-fixed and paraffin-embedded specimens of these cases were chosen for analysis. Clinical data were recorded, including age, sex, and Eastern Cooperative Oncology Group (ECOG) performance status (PS) score. Pathological variables were re-evaluated by 2 pathologists separately, including pT stage, Fuhrman grade and histological subtype. Patients were staged according to the 1997 staging system of the Union International Contre le Cancer (UICC) and American Joint Committee on Cancer (AJCC) [14]. The histological subtypes were stratified using Heidelberg classification [15] and the nuclear grade of tumors was determined by e Fuhrman grading scheme [16]. Performance status was determined using the Eastern Cooperative Oncology Group Performance Score (ECOG-PS) scale. The patients consisted of 329 men and 153 women, aging 19 to 94 years old, with a mean age of 62.1. There were 384 conventional, 81 papillary, and 17 chromophobe cases of RCCs respectively. 45 patients had distant metastases (to the lung in 12 patient, bone in 4 patients, the lymph nodes in 14 patients, gastrointestinal tract in 7 patients, soft tissue in 2 patients and adrenal gland in 6 patients) at the time of surgery. 324 (67.2%) tumors were discovered incidentally, 126 (26.1%) were locally symptomatic and 32 patients (6.6%) had systemic disease symptoms. The median period of follow-up for all patients was 62 months, ranging from 13 to 185 months.

### Construction of tissue microarray blocks and immunohistochemistry

For immunohistochemical evaluation, tissue microarrays were constructed using a manual tissue arrayer with accessory (Beecher, Silver Spring, MD). 3 cylindrical core biopsies (0.6 mm in diameter) were taken from different sites of each tumor and precisely arrayed in a recipient paraffin tissue microarray block. 10 specimens of non-neoplastic renal tissue resected from adjacent regions of renal cell carcinoma were also analysed for comparison.

The procedures of immunohistochemical studies were performed as described previously [13]. In brief, 4-mm tissue microarray sections were incubated with the mouse monoclonal antibodies targeting Skp2 (1:100, Zymed), Cks1 (1:250, Zymed), p27^Kip1 ^(1:50, Santa Cruz), using an automated immunostainer(Leica AutoStainer XL,). Binding of the primary antibody was assessed using the DAKO EnVision kit (DAKO Corp.), and the hematoxylin served as counterstain. Incubation without the primary antibody was used as a negative control.

### Assessment of immunohistochemical staining

Two pathologists, blinded to the clinicopathological data and patients' outcomes, independently evaluated immunoreactivity of the tissue microarray slides. Discrepancies were resolved by simultaneous re-examination of the slides by both investigators. Immunostaining was evaluated by recording the percentage of cells staining and scoring the area of maximum staining on a 4-point scale, with 0 representing no staining and 3 representing the highest staining. The results from 3 cores in the same patient were averaged to obtain a mean value for subsequent statistical analysis. According to the median percentage of positive cells for each antibody, the protein expression results were classified into 3 groups: Negative group; Low group (less than the median value) and High group (greater than the median value). The median values were: 40% for p27, 10% for Skp2, and 20% for Cks1. As described in previous studies [13], the expressions of Skp2 and Cks1 was further stratified as either positive or negative (Low+High group versus Negative group), while the p27^Kip1 ^expression was further classified as high expression (High group) and low expression (Negative+Low group).

### Statistical analysis

Statistical data analyses were performed using SPSS 11.0 statistical software package (SPSS Inc., Chicago, IL). First, the associations among Skp2, Cks1, p27^Kip1 ^and clinicopathological factors were explored using χ^2 ^test or Fisher's exact test as appropriate. Correlations between variables were tested according to the Spearman correlation test. Recurrence-free survival (RFS) and cancer-specific survival (CSS) were analyzed by Kaplan-Meier curves. The prognostic role of different parameters in the disease recurrence and patient death was analyzed with the log-rank test. For multivariate testing, a stepwise forward Cox's proportional hazards regression model was performed to define the risk factors for tumor recurrence and patient death. *P *values less than 0.05 were considered significant. Two-sided tests were used throughout all the analyses.

## Results

### Immunohistochemical expression and its correlation with clinicopathological variables

**Figure 1:**
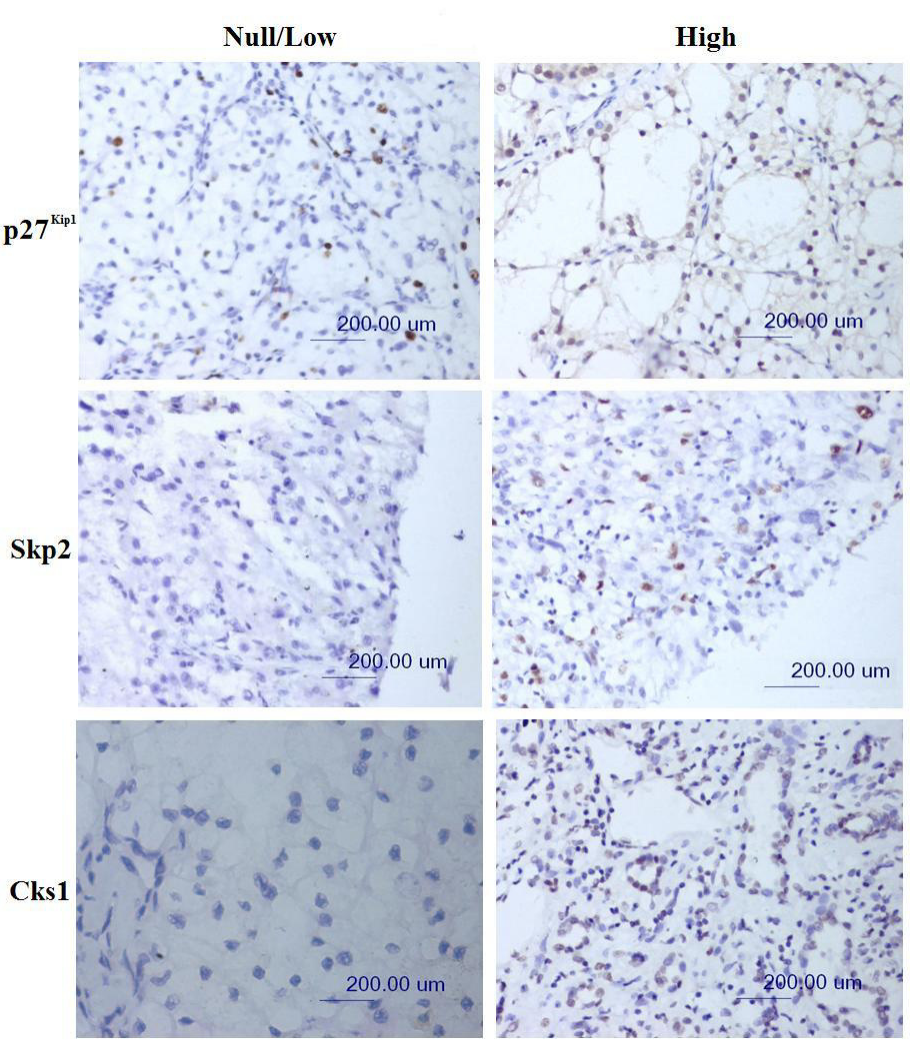
**Expression of p27^Kip1^, Skp2 and Cks1**. The proteins showing null/low or high levels of expression with nuclear pattern. (immunohistochemical staining; original magnification ×200; scale bar, 200 μm).

Representative examples of reactivity for p27^Kip1^, Skp2, and Cks1 are shown in Figure 1.

In nonneoplastic renal tissue, the distal tubules, glomerular epithelial cells, and pelvic urothelium showed strong nuclear p27^Kip1 ^immunoreactivity but lacked immunoreactivity for Skp2 and Cks1.

The protein expression and clinicopathological data of the patients are summarized in Table 1. As shown in Table 1, Skp2, Cks1, and p27^Kip1 ^expressions were not associated with patients' age or gender, as well as the ECOG-PS. In addition, none of the protein expression was associated with the grade.

**Table 1:** Expression of p27^Kip1^, Skp2, and Cks1 in relation to clinicopathological parameters

		**P27**			**Skp2**			**Cks1**		
				
Variables	No.	Low	High	p-value	N	P	p-value	N	P	p-value
Age, years										
Mean		62.5	61.3	0.904	62.0	65.1	0.723	60.0	62.3	0.630
Gender										
Men	329	213	116		281	48		272	57	
Women	153	101	52	0.785	130	23	0.898	120	33	0.266
Metastasis										
Negative	437	279	158		376	61		359	78	
Positive	45	35	10	0.062	35	10	0.136	33	12	0.148
Histology										
Clear cell	384	239	145		316	68		302	82	
Papillary	81	61	20		78	3		73	8	
Chromophobe	17	14	3	0.008^a^	17	0	<0.001^a^	17	0	0.003^a^
pT stage										
1	255	141	114		243	12		217	38	
2	103	78	25		83	20		78	25	
3+4	124	95	29	0.002^b^	85	39	<0.001^b^	97	27	0.304^b^
Tumor size										
≤ 7.0	324	188	136		298	26		273	51	
>7.0	158	126	32	<0.001	113	45	<0.001	119	39	0.018
Grade										
1	78	46	32		68	10		60	18	
2	250	159	91		215	35		201	49	
3	121	86	35		102	19		103	18	
4	33	23	10	0.075^c^	26	7	0.361^c^	28	5	0.149^c^
Necrosis										
Absent	432	271	161		376	56		352	80	
Present	50	43	7	0.001	35	15	0.001	40	10	0.799
ECOG-PS										
= 0	388	250	138		335	53		313	75	
>0	94	64	30	0.505	76	18	0.178	79	15	0.452

Note: N indicates negative; P indicates positive;

^a ^Histology, clear cell vs. others.

^b^Stage, Stages I and II vs. Stages III and IV.

^c ^Grade, Grade1/2 vs. Grade3/4.

P27^Kip1 ^expression was noted in 357 cases (74%), and high expression of p27^Kip1 ^was identified in 168 cases (37%). The expression of p27^Kip1 ^was significantly higher in clear-cell RCC compared with other histological types (P = 0.008). Furthermore, the expression significantly decreased with increasing tumor stage (P = 0.002) and tumor size (P < 0.001). In addition, the p27^Kip1 ^was also significantly downregulated in cases with tumor necrosis (P = 0.001). And the expression was not significantly different between primary and metastatic tumors (P = 0.062).

Skp2 expression was noted in 68 of 384 (18%) clear cell and 3 of 81 (4%) papillary tumors, but none of chromophobe tumors. Skp2 expression was significantly correlated with tumor stage (P < 0.001) and tumor size (P < 0.001), as well as tumor necrosis (P = 0.001). But Skp2 expression was not significantly different between primary and metastatic tumors (P = 0.136).

Cks1 expression was similar to Skp2, noted only in 82 of 384 (21%) clear cell and 8 of 81(10%) papillary tumors. Among various clinicopathological variables, the expression of Cks1 was only correlated with tumor size (P = 0.018).

### Associations among P27^Kip1^, Skp2 and Cks1 protein expression

A strong correlation was found between Skp2 and Cks1 expression (P < 0.001), both of which were inversely related to p27^Kip1 ^levels (P = 0.006 and P < 0.001). A further test was performed between patients with different metastatic status and histologic types. In primary RCCs, relation between Skp2 and Cks1 expression and their inverse relations to p27^Kip1 ^levels are statistically significant (P < 0.001, P = 0.001 and P = 0.001, respectively). But in metastatic RCCs, no correlation was found: 5 Skp2-positive RCCs showed Cks1 positive expression, while 5 Skp2-positive and 10 Cks1-positive RCCs showed low p27 expression, with a P value of 0.058, 0.179 and 0.893, respectively.

In clear-cell RCCs, the correlation among the protein expression was the same as that in the whole patients. But in papillary tumors, only the inverse associations between p27^Kip1 ^and Cks1 was statistically significant (P = 0.027).

### Survival analysis

At the end of follow-up, 131 patients died due to cancer progression, 36 patients died of unrelated causes, and 128 patients developed disease recurrence.

**Figure 2:**
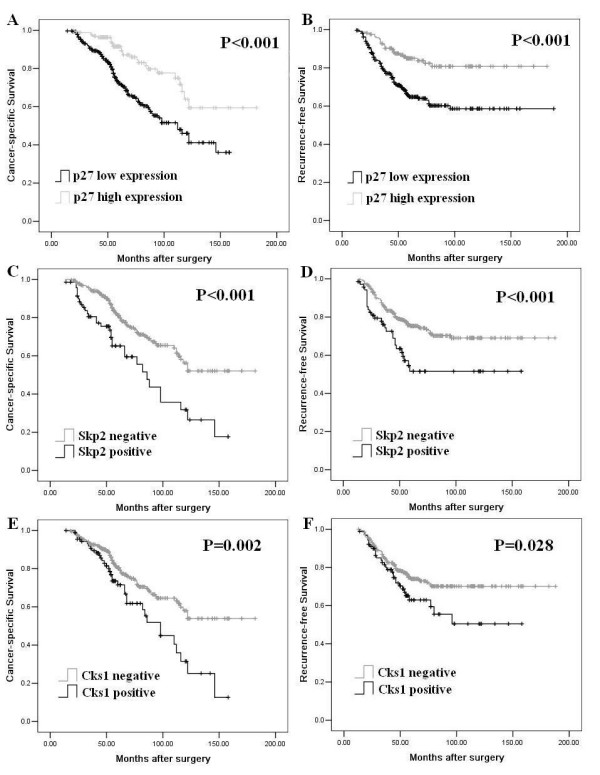
**Kaplan-Meier survival curves according to protein expression of p27^Kip1^, Skp2 and Cks1**. Cancer-specific survival of patients with RCC were significantly associated with the expression levels of p27^Kip1 ^(A), Skp2(C), Cks1(E); Recurrence-free survival were significantly associated with the expression levels of p27^Kip1 ^(B), Skp2(D), Cks1(F).

In the univariate analysis, the Kaplan-Meier survival curves showed that high Skp2 and Cks1 expression and low p27^Kip1 ^expression related to a poor survival with statistical significance, regardless of endpoints (Table 2, Fig. 2). The Cox's proportional hazards regression model proved that tumor stage, tumor size and low p27^Kip1 ^expression or lack of p27^Kip1 ^immunoreactivity were independent prognostic factors for different endpoints in patients with RCC (Table 2).

**Table 2:** Univariate and Multivariate Survival Analysis

	**P value on CSS**		**P value on RFS**	
		
Variables	Univariate^a^	Multivariate^b^	Univariate^a^	Multivariate^b^
Age	0.556	0.236	0.522	0.413
Gender	0.598	0.326	0.885	0.590
TNM stage	<0.001	<0.001	<0.001	<0.001
Tumor size	<0.001	<0.001	<0.001	<0.001
Grade	0.388	0.078	0.085	0.219
Histology	0.859	0.396	0.704	0.730
ECOG-PS	0.046	0.874	0.259	0.427
Necrosis	<0.001	0.117	<0.001	0.242
p27^Kip1^	<0.001	0.003	<0.001	0.002
Skp2	<0.001	0.360	0.001	0.966
Cks1	0.002	0.110	0.029	0.482

Note: OS: overall survival; CSS: cancer-specific survival; RFS: recurrence-free survival.

^a ^Statistical analyses were performed by the log-rank test.

^b ^Statistical analyses were performed by Cox proportional-hazards regression model.

## Discussion

To our knowledge, the current study presents the largest samples for investigating the expression of p27^Kip1 ^and its interacting cell cycle regulators: Skp2 and Cks1. This is also the first article to analyse Cks1 expression in renal cell carcinoma, with respect to possible associations with clinicopathological data as well as patients' prognosis. Furthermore, for the first time, the possible relation between levels of p27^Kip1 ^and of its specific ubiquitin ligase subunit Skp2 and Cks1 was assessed in renal cell carcinoma.

p27^Kip1 ^is a member of the Cip/Kip family of CDK inhibitory proteins and serves as an important regulator for both cellular proliferation and tissue differentiation. Till now, the significant association between low p27^Kip1 ^expression and adverse pathologic features or poor survival has been found in patients with a variety of neoplasms, including carcinoma of the gastrointestinal system [17], prostate [18,19], breast [11], and lung [12]. However, previous studies on p27^Kip1 ^expression in RCCs presented contradictory results. The low p27^Kip1 ^expression was found to be related to high-stage and high-grade RCCs by Langner C et al. [13] and Hedberg et al. [20]. Yet, other studies reported that the p27^Kip1 ^level only was only related to the TNM stage [21] or no associations at all [22]. Furthermore, although Hedberg et al. [20] reported the different p27^Kip1 ^expression patterns among various histologic subtypes, such results were not confirmed in other studies [13]. In our study, the expression of p27^Kip1 ^was significantly higher in clear-cell RCC compared with other histologic types and inversely related to TNM stage and tumor size. And no association between p27^Kip1 ^expression and metastatic status confirms the result in previous study for RCC [13] and other carcinomas [23].

More intriguingly, the controversy regarding association between grade and p27^Kip1 ^expression arised not only in RCCs, but also in many other cancers [24,25]. This discrepancy might be ascribed to the following reasons. First, the assessment of the grade may be highly observer dependent. Second, insufficient formalin fixation in some specimens may result in artificial reduced labeling of p27^Kip1 ^in TMA. More likely, the root cause might stem from the complex regulatory mechanisms of p27^Kip1 ^abundance. For instance, a new ubiquitin ligase for p27^Kip1 ^has recently been identified, which is a member for Skp2-independent down-regulation of p27^Kip1 ^at the G0–G1 transition [26].

The regulation of p27^Kip1 ^is mainly determined by its rate of degradation rather than by transcription or translation. Recent studies indicate that Skp2 and Cks1, the cell-cycle regulatory proteins, play essential roles in the degradation of p27^Kip1^. Skp2 is an ubiquitin ligase responsible for targeting P27^Kip1 ^to the proteasome and Cks1 is an essential cofactor for efficient Skp2 dependent p27^Kip1 ^ubiquitination. Several studies have provided evidence that both the Skp2 and Cks1 may have important roles in the development of tumor aggressiveness and its expression may be used as an independent prognostic factor for survival in various carcinomas[12,13,17,27]. In this study, Skp2 expression was found to be significantly correlated with advanced T stage, larger tumor size and necrosis, while Cks1 only correlated with tumor size. The results imply their importance in disease progression and confirm the previous studies. However, the different expression pattern among histologic types, such as no expression of Skp2 and Cks1 in chromophobe RCC, has never been previously reported. Thus, the expression of Skp2 and Cks1 combined with p27^Kip1 ^could help to separate the chromophobe from the clear cell subtype.

As for the association among p27^Kip1^, Skp2 and Cks1, it is still in dispute [10-13]. In this study, there was a significant positive correlation between SKP2 and CKS1 expression, as well as an inverse relationship between p27^Kip1 ^and the 2 proteins. To examine whether other factors affected the association, we carried out further subanalysis dividing patients into subgroups based upon histologic subtypes and metastatic status. The correlation among these proteins was only found to be significant in primary and clear-cell RCCs. The observation may partially explain the current dispute, as the different samples used by different researchers. It also suggests that p27^Kip1 ^downregulation may involve several pathways and occur through corresponding pathway in different status.

Consistent with previous studies [13,20-22], we found that TNM stage and tumor size were strong predictive prognostic markers in patients with RCC. With regard to prognostic significance of protein expression, our study also demonstrated that low p27^Kip1 ^expression significantly correlated with a poor outcome. Although the Skp2 and Cks1 expression correlated within inferior CSS and DFS in our univariate analysis, it did not remain prognostically independent in multivariate comparison.

## Conclusion

In summary, our study suggests that immunohistochemical expression levels of p27^Kip1^, Skp2 and Cks1 may serve as markers with prognostic implication. The inverse association between p27^Kip1 ^and Skp2 or Cks1 indicates the important roles of Skp2 and Cks1 in targeting the destruction of p27^Kip1^, and these regulatory proteins may well be considered as novel targets of therapeutic intervention in RCC in the future.

## Competing interests

The authors declare that they have no competing interests.

## Authors' contributions

ZL and QF performed experiments and wrote the manuscript. JL has made substantial contributions to conception and design, FW and KD has made substantial contribution to acquisition of the clinicopathological and follow-up data.
